# Randomized Controlled Trial of Cardiac Rehabilitation Using the Balance Exercise Assist Robot in Older Adults with Cardiovascular Disease

**DOI:** 10.3390/jcdd11050133

**Published:** 2024-04-25

**Authors:** Akihiro Hirashiki, Atsuya Shimizu, Takahiro Kamihara, Manabu Kokubo, Kakeru Hashimoto, Ikue Ueda, Kenji Sato, Koki Kawamura, Naoki Itoh, Toyoaki Murohara, Hitoshi Kagaya, Izumi Kondo

**Affiliations:** 1 Department of Cardiology, National Center for Geriatrics and Gerontology, Obu 474-8511, Japan; ashimizu@ncgg.go.jp (A.S.); kamihara@ncgg.go.jp (T.K.);; 2 Department of Cardiology, Nagoya University Graduate School of Medicine, Nagoya 466-8560, Japan; murohara@med.nagoya-u.ac.jp; 3 Department of Rehabilitation, National Center for Geriatrics and Gerontology, Obu 474-8511, Japanikueueda@ncgg.go.jp (I.U.); k-sato@ncgg.go.jp (K.S.); kawamura@ncgg.go.jp (K.K.); n-itoh@ncgg.go.jp (N.I.); hkagaya2@ncgg.go.jp (H.K.); ik7710@ncgg.go.jp (I.K.)

**Keywords:** balance exercise assist robot, older adults, cardiac rehabilitation, robotic rehabilitation

## Abstract

Background: Recent studies have investigated the effects of exercise on the functional capacity of older adults; training with a balance exercise assist robot (BEAR) effectively improves posture. This study compared the clinical safety and efficacy of training using BEAR video games to conventional resistance training in older adults with cardiovascular disease (CVD). Methods: Ninety patients (mean age: 78 years) hospitalized due to worsening CVD were randomized to cardiac rehabilitation (CR) Group R (conventional resistance training) or Group B (training using BEAR). After appropriate therapy, patients underwent laboratory testing and functional evaluation using the timed up-and-go test (TUG), short physical performance battery (SPPB), and functional independence measure (FIM) just before discharge and 4 months after CR. The rates of CVD readmission, cardiac death, and fall-related fractures were monitored. Results: BEAR had no adverse effects during exercise. At 4 months, TUG and SPPB improved significantly in both groups, with no significant difference between them. FIM motor and the Geriatric Nutritional Risk Index were significantly improved in Group B versus Group R. There was no significant difference in cardiac events and fall-related fractures between the two groups. Conclusion: CR with BEAR is safe and comparable to conventional resistance training for improving balance in older adults with CVD.

## 1. Introduction

Older adults now represent a growing sector of the population since they are living longer, and the world’s population is increasingly ageing [1]. However, scientific evidence for the chronic care of older adults is still lacking, and the assessment of and therapeutic intervention for frailty is a particularly urgent issue. Specifically, in addition to preventing the occurrence of cardiovascular events in patients with cardiovascular disease (CVD), it is necessary to prevent declines in the activities of daily living due to fractures caused by falls, which are particularly frequent in older adults. Falling is known to be influenced not only by muscle strength, but also by balance [2]. Therefore, as part of an industry–academic collaboration, we have developed the balance exercise assist robot (BEAR), a balance-focused exercise therapy, as an intervention for older adults with CVD and verified its effectiveness in preventing patient frailty, as reported elsewhere [3,4,5]. Briefly, BEAR is a stand-up-and-ride transport robot and personal mobility device with a two-wheel inverted balance. When the rider leans forward, the wheels rotate in the same direction, and the robot moves forward until the rider’s body returns to a vertical position [5].

The balance training device we are developing and supporting the demonstration of is characterized by the use of inverted pendulum control robot technology to facilitate the learning of postural control strategies. The motors are controlled to keep the user upright by constantly detecting the user’s posture with sensors.

Recent research has focused on the effects of on in improving the functional capacity of frail older adults [4,6]. Training with BEAR is effective in improving posture. However, little information is available regarding the safety and efficacy of BEAR during cardiac rehabilitation (CR) in older adults with CVD [4]. Therefore, the aim of the present study was to compare the clinical safety and efficacy of postural strategy training using the BEAR system with those of conventional resistance training during CR in older adults with CVD.

## 2. Materials and Methods

### 2.1. Study Population

This study was approved by the Nagoya University Certified Clinical Research Review Board and was registered with the Japan Registry of Clinical Trials (registration no. jRCTs042190086). This study was approved by the Ethics Review Board of Nagoya University approved the study (Approval no. 2020) and complied with the Declaration of Helsinki. Written informed consent was obtained from each participant.

**Figure 1:**
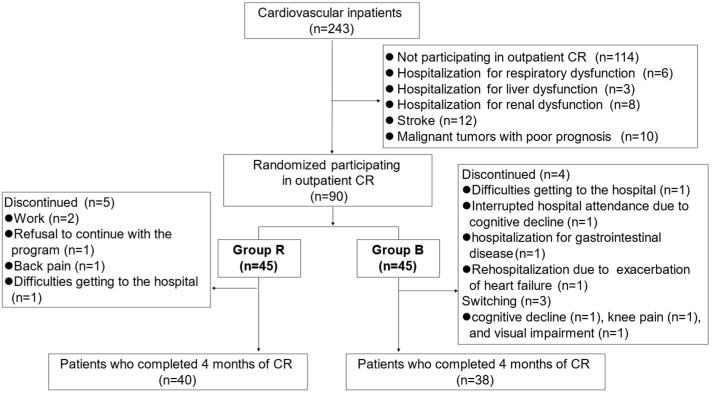
Flowchart of participant enrollment.

This prospective interventional study involved 243 patients who were at least 65 years old and who had been hospitalized for worsening CVD at the Department of Cardiology, National Center for Geriatrics and Gerontology (Obu, Japan), between December 2019 and December 2021. Figure 1 shows a flowchart of participant enrollment.

Participants performed a cardiopulmonary exercise test (CPX); underwent laboratory measurements, including GNRI, which is a screening tool used to assess nutritional status in older people and is primarily based on serum albumin and BMI [7], echocardiography, and a physical function evaluation; and completed questionnaires, including the Functional Independence Measure (FIM), Geriatric Depression Scale (GDS), and Japanese version of the Montreal Cognitive Assessment (MoCA-J), once their condition had stabilized after appropriate therapy.

Patients with structural heart disease, including coronary artery disease (i.e., having experienced angina pectoris or myocardial infarction, with a history of revascularization procedures); symptomatic heart failure (non-ischemic cardiomyopathy, ischemia, tachycardia, bradycardia, valvular disease, or hypertension); or other CVD (aortic disease, peripheral artery disease, and other vascular diseases), were eligible for inclusion in the study. Non-ischemic cardiomyopathies were defined as ventricular myocardial abnormalities in the absence of coronary artery disease or valvular, pericardial, or congenital heart disease [8]. Tachycardia and bradycardia included atrial, supraventricular, and ventricular arrhythmias, sick sinus syndrome, and atrioventricular block in the absence of structural heart disease. Valvular heart disease was diagnosed on the basis of hemodynamic or echocardiographic findings or a history of valvular or congenital cardiac surgery, according to the American College of Cardiology/American Heart Association guidelines [9]. Hypertension was defined as systolic blood pressure ≥140 mmHg, diastolic blood pressure ≥90 mmHg, or a history of treatment for hypertension. Worsening heart failure was defined as a clinical syndrome comprising symptoms and/or signs due to structural and/or functional cardiac abnormalities accompanied by elevated natriuretic peptide concentrations and/or objective evidence of pulmonary or systemic congestion [10].

Patients with severe respiratory dysfunction (i.e., receiving long-term oxygen therapy for respiratory disease), liver dysfunction (Child–Pugh score Class C), stroke, renal dysfunction (reduced glomerular filtration rate and albuminuria category G5), malignant tumors with a prognosis of <1 year, and visual or hearing impairments that could interfere with playing the video games were excluded from the study. Patients in whom CR was contraindicated as per the Guidelines for Rehabilitation in Patients with Cardiovascular Disease of the Japanese Circulation Society [11] and patients who did not understand how to operate the BEAR system were also excluded from the study. After applying both inclusion and exclusion criteria, we finally included 90 consecutive CVD patients (Figure 1).

### 2.2. Balance Exercise Assist Robot

**Figure 2:**
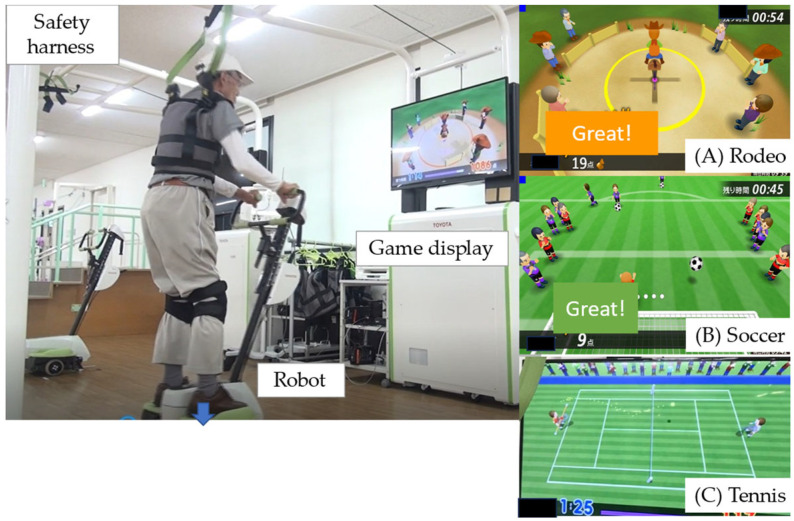
The balance exercise assist robot (BEAR) system. The BEAR moves backwards and forwards, as well as left and right, according to shifts in the operator’s center of gravity. These movements can be incorporated into balance exercises by playing three video games: (**A**) rodeo; (**B**) soccer; and (**C**) tennis.

The BEAR (Toyota Motor Corporation, Aichi, Japan) used in the present study had two wheels, an in-wheel motor controlled by an inverted pendulum system, and a foot plate on either side (Figure 2; Supplementary Video S1), as shown in our previous study [4]. The BEAR moves backwards and forwards and left and right according to shifts in the operator’s center of gravity. Balance exercise that promotes movement in these four directions is achieved by playing three video games, each of which focuses on different skills: (1) a skiing game, which requires left–right movement; (2) a rodeo game, which requires the operator to keep the robot stationary in the face of irregular disturbances; and (3) a tennis game, which involves forward–backward movement (Figure 2). The level of difficulty was adjusted to suit each individual, and the users performed the repetitive movements automatically [12]. To ensure safety during the exercises, we prepared a 2.4 m × 2.0 m space in the exercise room for the exclusive use of participants. The participants wore a safety harness to limit the risk of falls, but no lifting force was applied to reduce weight bearing.

### 2.3. CR Program

A 4-month prospective blinded randomized single-center trial was conducted. Randomization was stratified, with allocation adjusted for gender parity. To control for information bias, a prospective randomized open-label blinded end-point (PROBE) study design was used, with evaluations performed by physical therapists not involved in the study.

Examinations were performed when the patients were in a stable condition, after appropriate therapy for their disease. The intervention was performed on an outpatient basis for 1 h once a week for 4 months, for a total of 16 sessions. After 4 months, all patients were reassessed. The efficacy and safety of BEAR were tested in a randomized controlled trial (RCT) using the two-group randomization method described below. Patients were assigned at the start of CR and randomized 1:1 to either conventional and established resistance training (Group R) or BEAR (Group B). 

#### 2.3.1. Resistance Training Group (Group R)

Group R received resistance training as usual CR. Patients performed exercise (30 min aerobic exercise and 30 min resistance training), and were evaluated before and 4 months after the start of training. Resistance training was performed to improve muscular endurance and muscular strength by loading peripheral skeletal muscles and was adapted to individual muscular strength using body weight and tubes.

#### 2.3.2. BEAR Group (Group B)

Patients performed 30 min aerobic exercise using an ergometer plus 30 min balance exercises with the BEAR system. Each session of balance training with the BEAR system consisted of twelve episodes: four rounds of each of the three games, with each round lasting 1.5 min. 

### 2.4. Measurements

Peak oxygen uptake (VO_2peak_), gait speed (10 m gait test) the Short Physical Performance Battery (SPPB) score, timed up-and-go (TUG) time, and knee extensor strength were assessed just prior to discharge, when patients were medically stable, and at the end of the 4-month intervention period.

#### 2.4.1. Frailty

Frailty was defined according to the Japanese version of the Cardiovascular Health Study criteria (J-CHS) [13]. The J-CHS assesses five components, namely weight loss, physical activity, fatigue, muscle weakness, and gait speed, and it classifies people as being frail, pre-frail, or robust. Frailty is defined as the presence of signs or symptoms associated with at least three of the five components; pre-frailty is defined as the presence of signs or symptoms consistent with one or two components; and robust is defined as having no characteristics attributable to any of the components.

#### 2.4.2. Peak Oxygen Uptake

Each patient underwent a CPX (Minato Medical Science, Osaka, Japan) on a cycle ergometer at a progressively increasing work rate to maximum tolerance. The CPX was conducted in accordance with the recommendations of the American Thoracic Society and American College of Chest Physicians [14]. Gas exchange data were obtained breath by breath, and VO_2peak_ was determined as the highest oxygen uptake value recorded.

#### 2.4.3. Gait Speed

To determine gait speed, we used the 10 m gait test. Participants were instructed to walk twice along a 16 m walkway at a comfortable pace [15]. The time taken to cover 10 m along the walkway was measured and used to calculate gait speed.

#### 2.4.4. Short Physical Performance Battery

The SPPB evaluates lower limb function [16] through three components: balance tests (closed leg standing, semitandem standing, and tandem standing), walking time, and standing from a seated position. The reliability, validity, and feasibility of the SPPB in older adults have been reported elsewhere [17]. The maximum SPPB score is 12 points; the higher the score, the better the physical function.

#### 2.4.5. Timed Up-and-Go 

The TUG time includes the time required for a participant to stand up from an armchair, walk 3 m, turn, return to the chair, and sit down again [18].

#### 2.4.6. Muscle Strength of Knee Extension

The strength of knee extension was measured using a handheld dynamometer (µ-tas F-1; Anima, Tokyo, Japan). Knee extension was tested while the participant was seated, with the knee and hip flexed at 90° [19]. Each strength test was performed twice, and the best result was recorded.

#### 2.4.7. Functional Independence Measure

The FIM was developed to evaluate the rehabilitation of patients with disabilities, and it comprises two domains: motor and cognitive [20]. The motor domain (FIM motor) consists of 13 items: eating; grooming; bathing; dressing the upper body; dressing the lower body; toileting; bladder management; bowel management; transfer to bed, chair, or wheelchair; transfer to toilet; transfer to tub or shower; walking/wheelchair; and stairs. The cognitive domain (FIM cognitive) consists of five items: comprehension, expression, social interaction, problem solving, and memory. Items in both domains are scored from 1 to 7 (1, total assistance; 2, maximum assistance; 3, moderate assistance; 4, minimal contact assistance; 5, supervision; 6, modified independence; and 7, complete independence). The minimum total FIM score is 18 points, and the maximum is 126 points; minimum FIM motor and FIM cognitive scores are 13 and 5 points, respectively, and maximum FIM motor and FIM cognitive scores are 91 and 35 points, respectively. FIM scores were obtained by two physical therapists at discharge and 4 months.

#### 2.4.8. Bioelectrical Impedance Analysis

Body composition was assessed using multiple-frequency bioelectrical impedance analysis (S10; InBody, Seoul, Republic of Korea) using six different frequencies (1, 5, 50, 250, 500, and 1000 kHz). Patients remained supine on the bed with their arms and legs abducted, and reusable contact electrodes were placed on the first and third fingers of both hands and the medial and lateral sides of both ankles. The phase angle was calculated from the relationship between the resistance and reactance vectors based on bioelectrical impedance measurements. We used a resistance and reactance of 50 kHz to calculate the phase angle, and the phase angle in the whole body was used in the analyses. Evaluation of the phase angle has been shown to have high test–retest reliability and accuracy [21]. We also calculated the skeletal muscle index (SMI) by dividing the limb skeletal muscle mass (kg) by height squared (m^2^). The phase angle and SMI were measured before discharge and at 4 months.

### 2.5. Follow-Up

To evaluate whether the rate of cardiac events differed between patients in Groups R and B, we prospectively followed all patients for the occurrence of primary events, defined as cardiac death (from worsening congestive heart failure or sudden death) or unscheduled readmission for heart failure. Non-cardiac deaths were excluded from the analysis. The secondary endpoint was fall-related fractures.

### 2.6. Statistical Analysis

Continuous variables are reported as the mean ± SD, and categorical data are reported as percentages of all patients. Parameters obtained just before discharge after patients had been medically stabilized were compared with values obtained at the end of the 4-month intervention period using paired t-tests and Mann–Whitney U tests. The normality of the data distribution was confirmed using the Shapiro–Wilk test. The significance of differences between the two groups was assessed using the Chi-squared test for categorical variables and the Mann–Whitney rank-sum test for continuous variables. Cumulative cardiac event-free survival estimates were calculated using the Kaplan–Meier method, and the significance of differences was evaluated using a stratified log-rank test. All statistical analyses were performed using SPSS 17.0 (SPSS, Chicago, IL, USA). *p* < 0.05 was considered statistically significant.

## 3. Results

### 3.1. Baseline Characteristics

By June 2021, 90 patients were enrolled in the RCT; their clinical characteristics are presented in Table 1. The mean age was 78 years, 50% of patients were male, and the mean body mass index (BMI) was 22.7 kg/m^2^. There were no significant differences in age, sex, BMI, atrial fibrillation, coronary risk factors, underlying disease, medication use, echocardiographic data, CPX parameters, or frailty between Groups R and B (Table 1).

### 3.2. Safety and Continuity

There were no adverse effects during exercise recorded in either Group R or Group B, except for a decrease in blood pressure after using the BEAR system in one patient, which improved with intravenous fluid replacement. Five patients in Group R and four patients in Group B withdrew from the study, and three patients crossed over from Group B to Group R and were excluded from the analysis (Figure 1). The reasons for withdrawal from Group R were interruptions to CR due to work (*n* = 2), a refusal to continue with the program (*n* = 1), back pain (*n* = 1), and difficulties getting to the hospital (*n* = 1). The reasons for switching from Group B to Group R were an inability to follow instructions due to cognitive decline (*n* = 1), knee pain (*n* = 1), and visual impairment due to glaucoma resulting in screen blindness.

The reasons for withdrawal from Group B were difficulties getting to the hospital (*n* = 1), interrupted hospital attendance due to cognitive decline (*n* = 1), hospitalization for gastrointestinal disease (*n* = 1), and rehospitalization due to exacerbation of heart failure (*n* = 1).

### 3.3. Intra- and Intergroup Comparisons

**Figure 3:**
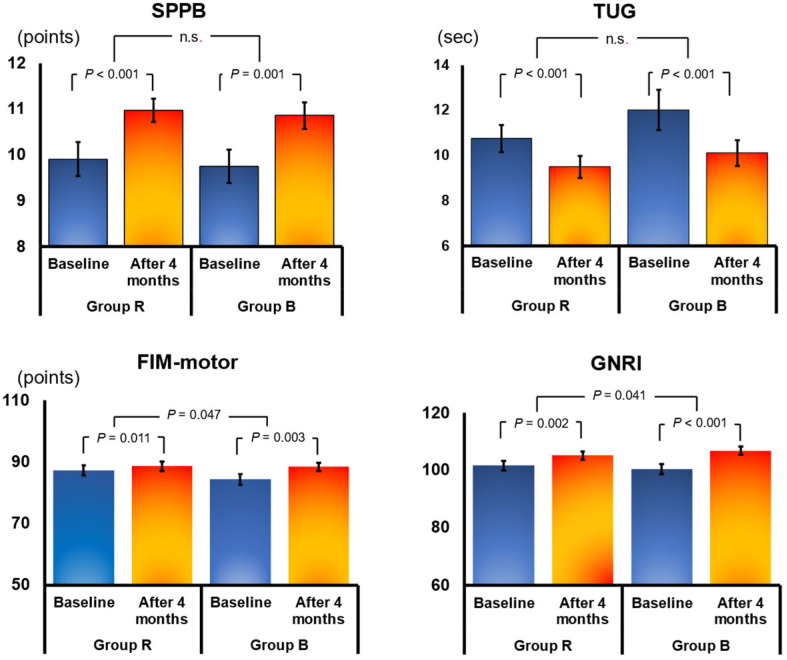
Effects of 4 months of cardiac rehabilitation (CR) in the resistance training group (Group R) and the group using the balance exercise assist robot (BEAR) system (Group B). Data show mean ± SE values at baseline and after 4 months. FIM motor, motor domain of the Functional Independence Measure; GNRI, Geriatric Nutritional Risk Index; SPPB, Short Physical Performance Battery; TUG, timed up-and-go test. n.s., no significant.

In all, 78 patients were included in the final analysis (39 each in Group B and Group R). The mean age of these 78 patients was 78 years, and 41% were male. Changes in parameters from discharge to 4 months and comparisons between the two groups are presented in Table 2. The Geriatric Nutritional Risk Index (GNRI), a nutritional index for older adults calculated from albumin, improved significantly from discharge to 4 months in both Group B (from 100.8 ± 11.1 to 107.3 ± 8.9; change 5.9 ± 6.4; *p* < 0.001) and Group R (from 102.5 ± 10.3 to 105.4 ± 9.9; change 3.3 ± 6.0; *p* = 0.001; Figure 3; Table 2). However, the change in GNRI was significantly better in Group B than in Group R (*p* = 0.041). Similarly, FIM motor improved significantly in both groups at 4 months, but the improvement was significantly better in Group B (*p* = 0.047; Figure 3; Table 2). There were no significant differences between Groups R and B in other parameters. There were no significant improvements at 4 months in B-type natriuretic peptide, estimated glomerular filtration rate (eGFR), echocardiography, and CPX VO_2peak_ in either group (Table 2). Conversely, significant improvements were noted in knee extensor strength, 10-m comfortable gate speed, TUG (a comprehensive test of gait and mobility), and SPPB (a measure of balance) at 4 months in both groups; however, there were no significant differences in the magnitude of changes between the two groups (Figure 3; Table 2).

### 3.4. Event-Free Survival

**Figure 4:**
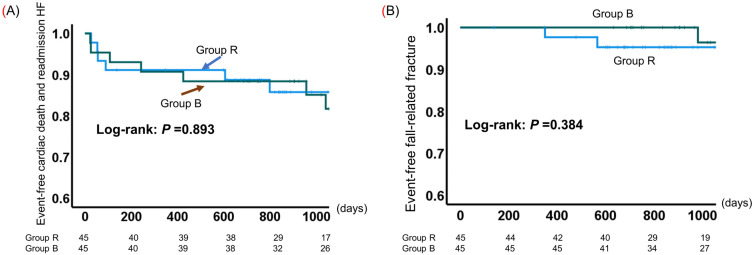
Kaplan–Meier estimates of event-free survival in older adults with cardiovascular disease (CVD) in the resistance training group (Group R) and the group using the balance exercise assist robot (BEAR) system (Group B). (**A**) Cardiac death and readmission for heart failure; (**B**) fall-related fractures.

The cumulative probability of event-free survival is shown in Figure 4. All patients were followed for a mean of 867 days (range of 28–1623 days) from the time of enrollment to the time of a cardiac event or the last assessment of survivors. Cardiac deaths were recorded for two patients each in Groups R and B. Nine patients in Group R and eight patients in Group B were rehospitalized for heart failure. The probability of event (i.e., cardiac death and heart failure readmission)-free survival was comparable between Groups R and B (log-rank test *p* = 0.893; Figure 4A). Although there were no fall-related fractures in Group B over the 2-year follow-up period, three patients in both groups eventually developed fractures; there was no significant difference in the incidence of fall-related fractures between the two groups (Figure 4B). 

## 4. Discussion

To the best of our knowledge, this is the first RCT to show the beneficial effects of CR with BEAR versus conventional CR with established resistance training in older adults with CVD. We also compared the incidence of cardiac events and fall-related fractures between the two groups. The main outcome of this study is the demonstrated safety and benefits of BEAR compared with resistance training in older patients with CVD, specifically concerning the following:

- In terms of safety, there were no accidents during exercise sessions in the BEAR group, and the exercises were safe for patients with heart disease. No fractures due to falls were observed during the follow-up period of up to 2 years.
- The dropout rate over a 4-month CR period was comparable between the BEAR and resistance training groups, so there were no problems with continuity.
- The effects of CR at 4 months were similar in the BEAR and resistance training groups. The improvements in FIM motor and GNRI at 4 months were significantly better in the BEAR than resistance training group.

### 4.1. Safety of BEAR

No adverse effects of using the BEAR system were seen. Nine percent of patients dropped out of the BEAR group, compared with 11% in the resistance training group. The reasons for dropping out of the BEAR group or switching to the resistance training group included visual or cognitive impairment or hospitalization. The use of the BEAR system appeared to be comparable to the use of conventional CR in terms of safety and adherence.

The risk of falls and trauma during exercise was considered a disadvantage of the BEAR system, but sufficient safety precautions were taken, such as the use of a safety harness while patients were using the BEAR system, so this is no longer considered a problem. There were no reports of adverse events, including falls or device malfunction, by patients using the BEAR system in this study, and we believe that we have demonstrated the safety of the system in patients with cardiac disease as long as their disease is under control.

### 4.2. Efficacy of BEAR

Although there are numerous reports of the effectiveness of rehabilitation with BEAR [3,5,10,19,20,21], there are none in patients with cardiac disease, except in our previous study, which was a single arm without a control group [4]. In the present study, CR with resistance training and using BEAR resulted in significant improvements in nutrition, physical function, and balance after 4 months, with no significant differences in the magnitude of the changes between the resistance training and BEAR groups, except for FIM motor and GNRI.

#### 4.2.1. Improvements in FIM motor

The improvement in FIM motor after CR for 4 months using the BEAR system was significantly greater than that seen with resistance training, but there was no difference between the groups in terms of improvements in FIM cognitive. Previous studies have reported that older adults with heart failure have reduced physical function (e.g., mobility and balance), reduced exercise tolerance due to complications such as frailty, and a higher incidence of falls [22] and orthopedic fractures [23] than healthy community-dwelling people. Exercise therapy that improves balance and mobility in addition to aerobic and resistance training is considered necessary, but the content of exercise therapy and its effectiveness for older people with heart failure have not been adequately tested.

#### 4.2.2. Improvements in GNRI

In the present study, the improvement in GNRI was significantly greater in the BEAR than the resistance training group. GNRI is a screening tool used to assess nutritional status in older people and is primarily based on serum albumin and BMI [7]. Heart failure with low GNRI is associated with increased all-cause mortality [24]. The systemic proinflammatory state is a major contributor to the cardiac pathology of heart failure, resulting in irreversible cardiac damage [25], although the underlying mechanism remains unclear. According to questionnaire respondents, BEAR is an enjoyable rehabilitation strategy [5], and we speculate that BEAR may improve a patient’s mental state and motivation, thus leading to an improvement in a patient’s diet.

### 4.3. BEAR vs. Resistance Training

Current clinical guidelines strongly recommend CR in patients with chronic heart failure [26], and the benefits of CR are well established, particularly in older adults with CVD, for whom a combination of aerobic and resistance training is recommended. A recent multicenter retrospective cohort study of patients with heart failure has found that CR is effective in both heart failure with preserved left ventricular ejection fraction and frailty [6]. The use of a BEAR has been shown to improve gait speed, TUG, and knee extension muscle strength in community-dwelling frail and pre-frail older adults [3]. In the present study, in which 34% of patients were frail and 36% had atrial fibrillation, CR with BEAR was demonstrated to be safe and comparable to conventional resistance training for improving balance in older adults with CVD who were medically stable and prescribed standard medications for their CVD.

The physical function and balance outcomes following CR with BEAR were comparable to those for CR with resistance training, demonstrating that exercise training with the BEAR system is safe and effective even for older adults with CVD. In addition, the incidence of cardiac events and fall-related fractures over the longer term (2 years) was comparable between CR with BEAR and CR with established resistance and aerobic training groups. Thus, we have demonstrated that the use of a BEAR system is comparable to conventional resistance training in improving exercise capacity and balance in older adults and frail patients with CVD.

### 4.4. Study Limitations

The limitations of this study include the small number of participants and the fact that it was performed at a single center. To confirm whether BEAR is more effective than resistance training in any respect, a larger number of cases need to be accumulated, along with performing subanalyses (e.g., according to age and sex). In the present study, no adverse events were reported during CR in either group, suggesting that this study can be safely replicated in older adults with heart failure. Further studies are needed to determine whether the effects of CR with BEAR differ in different subgroups.

## 5. Conclusions

Cardiac rehabilitation with the balance exercise assist robot was safe and without adverse events, but required a certain level of visual acuity and cognitive function. Cardiac rehabilitation that focuses on balance to prevent falls may be as effective as resistance training and may be useful for older adults with cardiovascular disease.

## Figures and Tables

**Table 1:** Clinical characteristics of the study participants.

	Group R (*n* = 45)	Group B (*n* = 45)	*p*-Value
Age (years)	79 ± 6	78 ± 6	0.633
Male sex	23 (51)	22 (49)	0.835
Body mass index (kg/m^2^)	22.6 ± 3.8	22.7 ± 3.2	0.931
Atrial fibrillation (%)	31.1	40.9	0.359
Coronary risk factor			
Hypertension	30 (67)	25 (56)	0.285
Diabetes	13 (29)	8 (18)	0.217
Dyslipidemia	22 (49)	17 (38)	0.293
Underlying diseases			
Worsening heart failure	32 (71)	35 (78)	0.512
Bradycardia (*n*)	6	4	
Non-ischemic cardiomyopathy (*n*)	3	3	
Ischemic heart disease (*n*)	4	6	
Tachycardia (*n*)	7	10	
Valvular disease (*n*)	6	8	
Hypertension (*n*)	5	4	
Other	1	0	
AMI	6 (13)	3 (7)	
AP	7 (16)	7 (16)	
Medications			
Diuretics	17 (38)	20 (44)	0.724
Tolvaptan	2 (4)	6 (13)	0.526
ACE-I/ARB	21 (47)	18 (40)	0.142
Beta-blocker	26 (58)	21 (47)	0.529
Spironolactone	6 (13)	8 (18)	0.297
Anticoagulant	14 (31)	19 (42)	0.566
Antiplatelet agent	23 (51)	20 (44)	0.279
Echocardiography			
Left atrial dimension (cm)	3.9 ± 0.7	4.0 ± 1.0	0.594
CPX			
Respiratory exchange ratio	1.14 ± 0.07	1.15 ± 0.07	0.294
Frailty (*n*)			
KCL (robust/pre-frail/frail)	10/20/15	15/14/16	0.351
J-CHS (robust/pre-frail/frail)	0/20/25	1/22/22	0.526

Unless indicated otherwise, data are given as the mean ± SD or *n* (%). ACE-I/ARB, angiotensin-converting enzyme inhibitor/angiotensin II receptor blocker; AMI, acute myocardial infarction; AP, angina pectoris; CPX, cardiopulmonary exercise test; Group B, cardiac rehabilitation using the balance exercise assist robot (BEAR) system; Group R, cardiac rehabilitation with resistance training; J-CHS, Japanese version of the Cardiovascular Health Study criteria; KCL, Kihon checklist.

**Table 2:** Changes in parameters from baseline to after 4 months of cardiac rehabilitation.

	Group R	*p-* Value	Group B	*p*-Value	*p*-Value (Group R vs. Group B)
**Albumin** (g/dL)					
Baseline	4.0 ± 0.4	<0.001	3.9 ± 0.5	<0.001	0.201
4 months	4.2 ± 0.3		4.2 ± 0.4		0.884
Change	0.2 ± 0.4		0.3 ± 0.4		0.122
**Total cholesterol** (mg/dL)					
Baseline	178.8 ± 38.0	0.027	169.9 ± 37.8	<0.001	0.269
4 months	190.6 ± 40.2		187.7 ± 45.7		0.753
Change	11.5 ± 33.9		17.8 ± 29.3		0.351
**BNP** (pg/dL)					
Baseline	118.1 ± 191.5	0.535	141.8 ± 185.4	0.649	0.553
4 months	107.6 ± 130.6		150.1 ± 172.1		0.203
Change	–17.4 ± 179.5		9.9 ± 142.2		0.436
**eGFR** (mL/min/1.73 m^2^)					
Baseline	53.6 ± 14.4	0.706	61.0 ± 16.5	0.064	0.025
4 months	53.3 ± 15.7		58.0 ± 13.9		0.131
Change	–0.4 ± 7.8		–3.0 ± 10.5		0.199
**GNRI**					
Baseline	102.5 ± 10.3	0.002	100.8 ± 11.1	<0.001	0.464
4 months	105.4 ± 9.9		107.3 ± 8.9		0.360
Change	3.3 ± 6.0		5.9 ± 6.4		0.041
**LVEF** (%)					
Baseline	57.5 ± 11.7	0.488	57.2 ± 14.1	0.393	0.916
4 months	58.1 ± 10.7		57.3 ± 12.3		0.758
Change	0.6 ± 5.2		–0.8 ± 6.3		0.270
**E/e′**					
Baseline	15.0 ± 6.2	0.328	14.2 ± 5.8	0.890	0.550
After 4 months	13.8 ± 5.8		13.8 ± 4.4		0.998
Change	–1.7 ± 7.9		–0.3 ± 7.2		0.390
**VO_2_ at AT** (mL/min/kg)					
Baseline	9.8 ± 1.6	0.037	9.4 ± 1.9	0.057	0.298
After 4 months	10.2 ± 2.1		9.9 ± 1.9		0.480
Change	0.2 ± 2.1		0.5 ± 1.6		0.451
**VO_2peak_** (mL/min/kg)					
Baseline	12.6 ± 2.6	0.052	12.4 ± 3.2	0.157	0.728
4 months	13.2 ± 3.1		13.0 ± 3.2		0.236
Change	1.3 ± 1.8		0.5 ± 2.1		0.080
**% VO_2peak_**					
Baseline	55.1 ± 12.2	0.227	55.2 ± 14.3	0.815	0.988
After 4 months	84.1 ± 143.2		57.2 ± 14.1		0.256
Change	29.0 ± 145.4		0.2 ± 10.3		0.232
**J-CHS score**					
Baseline	1.6 ± 0.5	0.003	1.5 ± 0.5	0.001	0.425
4 months	1.2 ± 0.5		1.1 ± 0.6		0.417
Change	–0.3 ± 0.6		–0.4 ± 0.7		0.737
**SPPB**					
Baseline	9.9 ± 2.4	<0.001	9.8 ± 2.3	0.001	0.865
4 months	11.0 ± 1.6		10.8 ± 1.8		0.702
Change	1.2 ± 1.6		0.9 ± 1.6		0.372
**TUG** (s)					
Baseline	10.8 ± 4.0	<0.001	12.4 ± 5.8	<0.001	0.130
4 months	9.5 ± 3.2		10.1 ± 3.7		0.418
Change	–1.1 ± 1.7		–1.9 ± 3.1		0.191
**Gait speed** (m/s)					
Baseline	1.0 ± 0.3	<0.001	1.0 ± 0.3	<0.001	0.971
4 months	1.2 ± 0.3		1.2 ± 0.3		0.643
Change	0.1 ± 0.2		0.2 ± 0.2		0.635
**Knee extension** (kgf)					
Baseline	22.7 ± 9.6	0.001	24.0 ± 10.1	0.011	0.527
4 months	25.9 ± 10.2		26.6 ± 10.5		0.770
Change	3.0 ± 5.5		2.4 ± 5.6		0.607
**FIM motor**					
Baseline	87.3 ± 5.5	0.011	84.3 ± 9.5	0.003	0.068
4 months	88.6 ± 4.6		88.4 ± 5.6		0.854
Change	1.5 ± 3.6		4.2 ± 8.3		0.047
**FIM cognitive**					
Baseline	34.2 ± 2.0	0.217	34.7 ± 0.9	0.183	0.121
4 months	34.5 ± 1.4		34.8 ± 0.6		0.118
Change	1.8 ± 4.2		4.4 ± 8.3		0.548
**GDS score**					
Baseline	4.1 ± 3.4	0.002	3.8 ± 3.9	0.028	0.618
4 months	3.3 ± 3.2		2.9 ± 3.3		0.609
Change	–1.0 ± 2.0		–1.1 ± 3.0		0.964
**MoCA-J score**					
Baseline	22.8 ± 3.9	0.148	22.5 ± 4.5	0.027	0.696
4 months	23.4 ± 3.9		23.8 ± 3.2		0.635
Change	1.2 ± 4.5		1.6 ± 4.6		0.706
**SMI** (kg/m^2^)					
Baseline	6.2 ± 1.2	0.027	6.3 ± 1.1	0.047	0.696
4 months	6.2 ± 1.0		6.6 ± 1.1		0.147
Change	–0.3 ± 2.4		0.2 ± 0.7		0.136

Unless indicated otherwise, data are given as the mean ± SD. AT, anaerobic threshold; BNP, B-type natriuretic peptide; E/e′, ratio of transmitral Doppler early filling velocity to tissue Doppler early diastolic mitral annular velocity; eGFR, estimated glomerular filtration rate; FIM cognitive, cognitive domain of the Functional Independence Measure; FIM motor, motor domain of the Functional Independence Measure; GDS, Geriatric Depression Scale; GNRI, Geriatric Nutritional Risk Index; Group B, cardiac rehabilitation using the balance exercise assist robot (BEAR) system; Group R, cardiac rehabilitation with resistance training; J-CHS, Japanese version of the Cardiovascular Health Study criteria; LVEF, left ventricular ejection fraction; MoCA-J, Japanese version of the Montreal Cognitive Assessment; SMI, skeletal muscle mass index; SPPB, Short Physical Performance Battery; TUG, timed up-and-go test; VO_2peak_, peak oxygen consumption.

## Data Availability

Data are contained within the article and Supplementary Materials.

## Supplementary Materials

The following supporting information can be downloaded at https://www.mdpi.com/article/10.3390/jcdd11050133/s1, Supplementary Video S1. Representative video showing a patient using the balance exercise assist robot (BEAR) system.
